# Complete genome sequence of *Chlamydia psittaci* NRM_5 strain isolated from the fecal samples of a wild Indian ring-necked parakeet (*Psittacula krameri manillens*is) in Japan

**DOI:** 10.1128/mra.01169-23

**Published:** 2024-04-30

**Authors:** Yukiko Sassa-O'Brien, Chien-Fu Wu, Yasuhiko Matsushita, Kenji Ohya, Hiromitsu Moriyama, Hideto Fukushi

**Affiliations:** 1 Laboratory of Veterinary Infectious Disease, Faculty of Agriculture, Tokyo University of Agriculture and Technology, Fuchu, Tokyo, Japan; 2 Laboratory of Molecular and Cellular Biology, Faculty of Agriculture, Tokyo University of Agriculture and Technology, Fuchu, Tokyo, Japan; 3 Gene Research Center, Tokyo University of Agriculture and Technology, Fuchu, Tokyo, Japan; 4 Laboratory of Veterinary Microbiology, Faculty of Applied Biological Sciences, Gifu University, Gifu, Gifu, Japan; University of Notre Dame, Notre Dame, Indiana, USA

**Keywords:** *Chlamydia psittaci*, ring-necked parakeet, allocated bird pathogen, ST-35

## Abstract

We report here the whole-genome sequence of the *Chlamydia psittaci* NRM_5 strain isolated from the fecal samples of wild Indian ring-necked parakeet (*Psittacula krameri manillensis*) in Japan. The sequence type is ST35, which is known to be associated with pigeons and doves, indicating the potential for transmission among bird species.

## ANNOUNCEMENT

The obligate intracellular Gram-negative bacterium *Chlamydia psittaci* is a zoonotic agent transmitted from birds to human that causes psittacosis. The etiological agent is secreted in bird feces and serves as a source of infection (1). We report the whole-genome sequence of a *C. psittaci* NRM_5 strain isolated from fecal sample of healthy wild Indian ring-necked parakeet (*Psittacula krameri manillensis*) in allochthonous flocks in urban areas of Japan in 2022. These parakeets, a successful invasive species in over 35 countries (2), have formed large flocks exceeding 1,000 birds in urban areas of Japan.

Sample from Nerima-ku, Tokyo, was processed to isolate *C. psittaci* using HeLa cells as described previously (3). The supernatant of infected HeLa cells, treated with DNaseI and RNaseA, was subjected to DNA extraction using QIAamp DNA mini kit (QIAGEN, Venlo, Netherlands). A DNA library was prepared with the MGIEasy FS DNA Library Prep Set (MGI Tech Japan, Tokyo, Japan), wherein enzymatically fragmented DNA was size-selected to approximately 300 base pairs using magnetic beads. Sequencing was performed on a DNBSEQ-G400RS sequencer (MGI Tech Japan, Tokyo, Japan) using a DNBSEQ-G400 High-throughput Sequencing Set FCL flow cell (MGI Tech Japan, Tokyo, Japan) with 2 × 150 bp paired-end protocol, outputting 37,638,078 reads and 11.291 Gbp.

Quality control and trimming were conducted with FastQC v.0.12.1 (4) and Trimmomatic v.0.27 (5). The trimmed reads were mapped to reference genomes of 27 chromosomes and 18 plasmids (Table 1) using Bowtie 2 v.2.5.2 (6). Aligned reads were assembled using SPAdes v.3.15.5 (7) with the --careful option and three chromosome contigs (786,574, 381,104, and 719 bp) and a plasmid contig (7,630 bp) were obtained, then the chromosome contigs were manually assembled. The assembled chromosome and plasmid sequences were mapped and polished using BWA-MEM v.0.7.17 (8), Pilon v.1.24 (9), Bowtie 2 v.2.5.2 (6), and Consed v.29.0 (10). Default parameters were used for all genome assembly tools unless otherwise specified. We confirmed the circularity of our sequences manually.

**TABLE 1:** Reference sequences using in this study

Strain	Accn. no. of chromosomal sequence	Accn. no. of plasmid sequence
*Chlamydia psittaci*		
6BC	CP002549	CP002550
CP3	CP003797	CP003813
MN	CP003792	CP003815
01DC12	HF545614	HF545615
Mat116	CP002744	‒^*a*^
WC	CP003796	CP003818
WS/RT/E30	CP003794	CP003819
Ful127	CP033059	CP033060
Horse_pl	CP025423	CP025424
BF_amazon_parrot_13	CP110211	CP110212
8882_placenta	CP092197	CP092198
9945_foetus	CP092199	CP092200
84/55	CP003790	CP003812
08DC60	CP002807	‒
02DC15	CP002806	‒
01DC11	CP002805	‒
C19/98	CP002804	‒
AMK	CP047319	CP047320
BL-84	CP094377	‒
GR9	CP003791	‒
VS225	CP003793	CP003817
CPS-QD/LS	CP103952	CP103953
*Chlamydia abortus*		
84/2334	CP031646	CP031647
15–58d44	OU508367	OU508368
LLG	CP018296	
*Chlamydia buteonis*		
SWA	CP067334 CP067334	‒
*Chlamydia* sp.		
Rostinovo-70	CP041038	CP041039

^*a*^ No plasmid sequence.

The chromosomal genome of NRM_5 was found to be 1,168,809 bp in size. The annotation was performed by DFAST (11), found 988 and 7 coding sequences in the chromosome and plasmid, respectively. The GC content of strain NRM_5 is 39.1%, consistent with known *Chlamydiaceae* (12–14), and three rRNAs and 39 tRNAs existed. The plasmid genome is 7,630 bp with 32.9% of GC content. The genome coverages were 8,390× and 7,430× for chromosome and plasmid construction, respectively.

The multilocus sequence typing, utilizing seven genes (*gatA*, *oppA*, *hflX*, *gidA*, *enoA*, *hemN*, and *fumC*) (15), assigned the *C. psittaci* NRM_5 to sequence type (ST) 35 using a database hosted at http://pubmlst.org/chlamydiales/. Since ST35 is a genotype associated with pigeons and doves (16, 17), the possibility of transmission among bird species is indicated.

## Data Availability

The genome sequences of NRM_5 strain has been deposited at DDBJ/EMBL/GenBank under the accession numbers AP028988 and AP028989 for chromosome and plasmid, respectively. The raw reads have been deposited under SRA number DRP010692, and the BioProject number is PRJDB16848.
